# Supplementary material for: Changes in Quality Characteristics and Metabolite Composition of Low-Temperature and Nitrogen-Modified Atmosphere in Indica Rice during Storage
Source: Foods. 2024 Sep 19;13(18):2968. doi: 10.3390/foods13182968 (PMC11431329; doi:10.3390/foods13182968)
Supplement: Supplementary file 1 [file foods-13-02968-s001.zip › foods-3186516-supplementary.pdf]

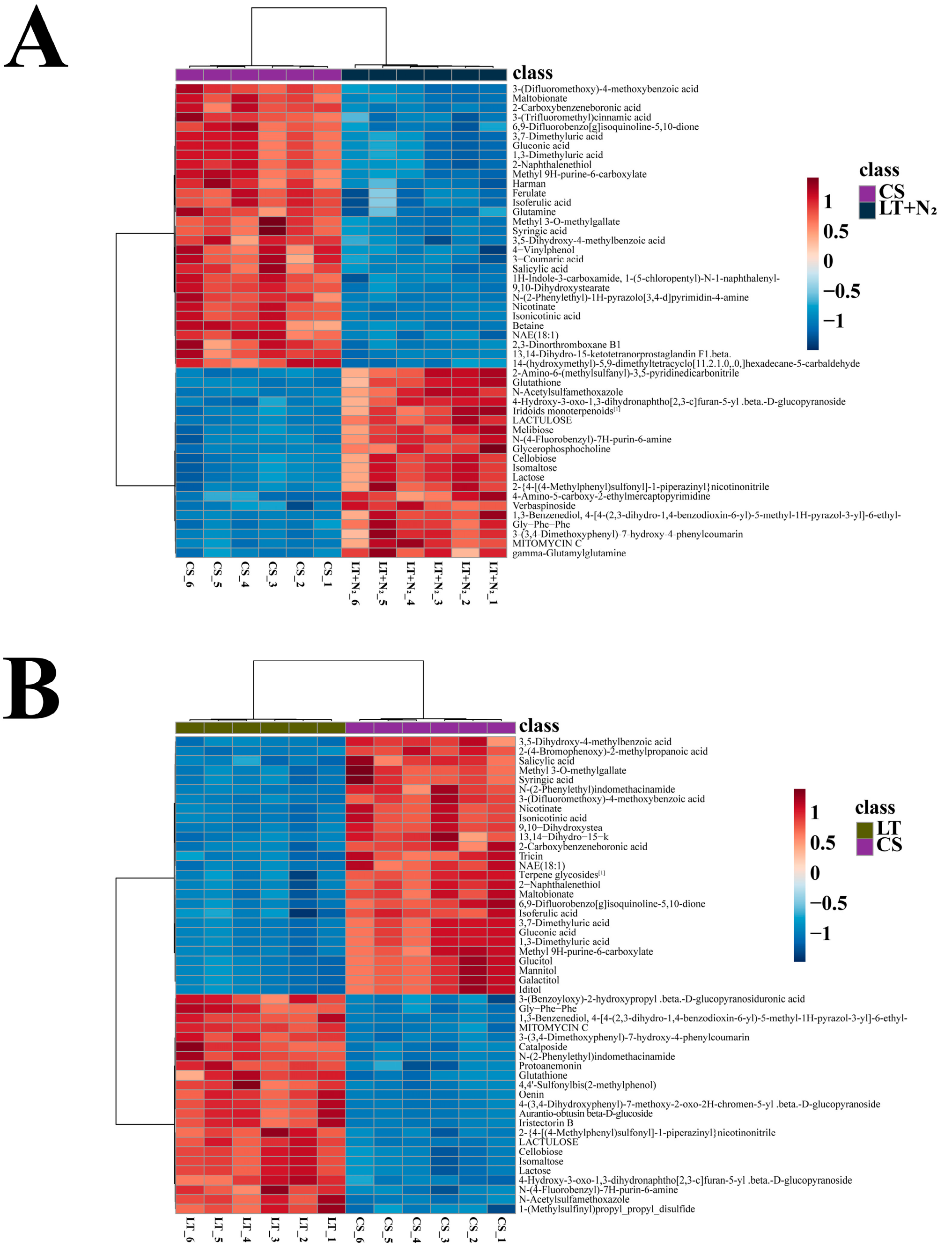

Figure S1. (A) The heat map of significantly differential metabolites in CS vs LT+N_2. (B) The heat map of significantly differential metabolites in CS vs LT. (C) The heat map of significantly differential metabolites in CS vs N_2. (D) The heat map of significantly differential metabolites in CS, N_2, LT, and LT+N_2. The colored blocks at different positions show the correlation coefficients between the metabolites at those positions. The colors red and blue are used to indicate positive and negative correlations respectively, with darker shades indicating stronger correlations.

Notes: Iridoids monoterpenoids [1]: (1aS,1bS,2S,5aR,6S,6aS)-6-((6-Deoxy-3-O-((2E)-3-(4-hydroxy-3-methoxyphenyl)prop-2-enoyl)-.alpha.-L-mannopyranosyl)oxy)-1a-(hydroxymethyl)-1a,1b,2,5a,6,6a-hexahydrooxireno[4,5]cyclopenta[1,2-c]pyran-2-yl.beta.-D-glucopyranoside.Terpene glycosides [1]: 5,7-Dihydroxy-1-((2-O-((2E)-3-(4-hydroxyphenyl)prop-2-enoyl)hexopyranosyl)oxy)-7-methyl-1,4a,5,6,7,7a-hexahydrocyclopenta[c]pyran-4-carboxylic acid)

C

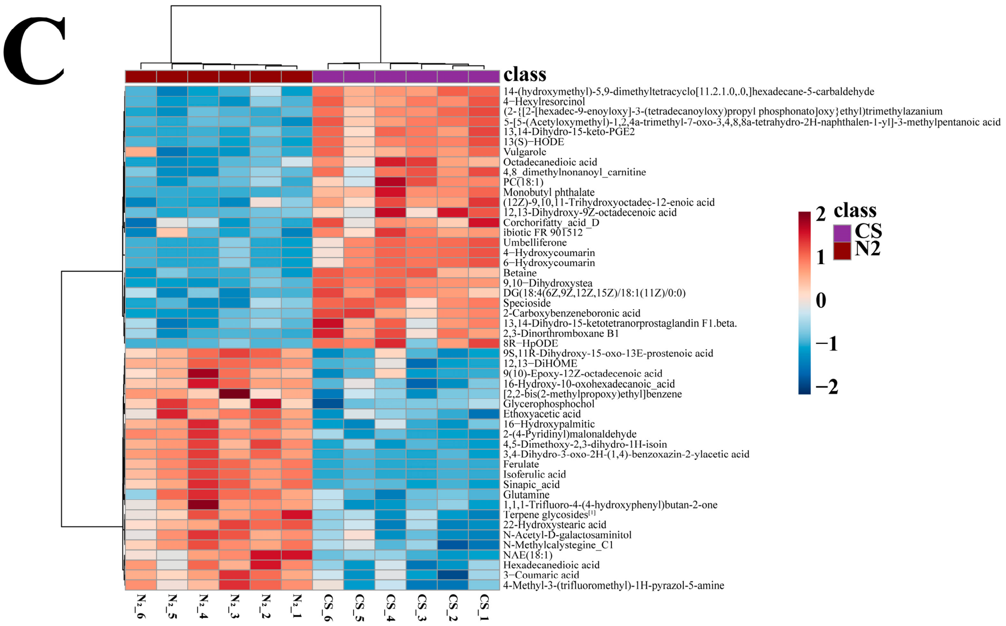

D

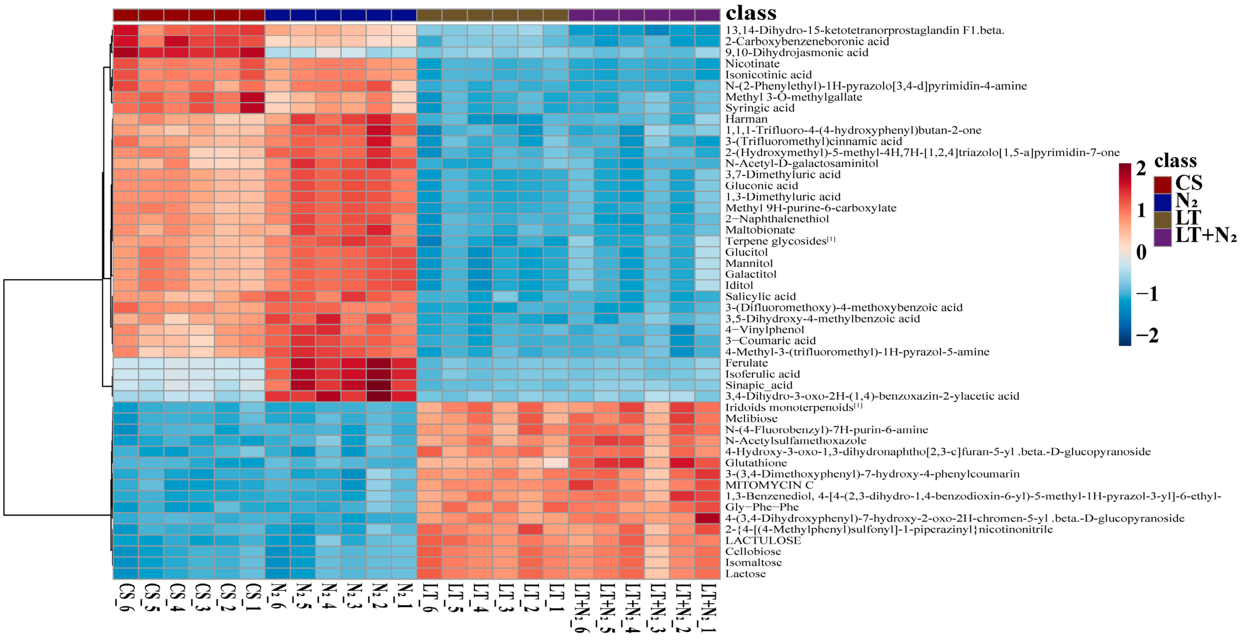

Figure S1. (continued).

Compound Name	CS vs LT+N_2			CS vs LT			CS vs N_2		
	VIP	P	UP/DOWN	VIP	P	UP/DOWN	VIP	P	UP/DOWN
Syringic acid	1.95	4.15E-08	DOWN	1.95	3.77E-08	DOWN	1.94	4.42E-03	DOWN
Salicylic acid	1.95	3.90E-08	DOWN	1.95	5.52E-08	DOWN	1.74	1.43E-02	UP
13(S)-HODE	1.78	1.04E-04	DOWN	1.44	6.69E-03	DOWN	2.49	2.81E-05	DOWN
2-Hydroxyglutaric acid	1.79	7.53E-05	DOWN	1.89	4.56E-06	DOWN	2.25	1.28E-04	UP
Isoferulic acid	1.94	2.43E-08	DOWN	1.92	1.08E-05	DOWN	2.53	2.24E-05	UP
2-Hydroxypalmitic acid	1.23	3.51E-02	DOWN	1.71	2.51E-03	DOWN	1.53	4.41E-02	DOWN
Hexadecanedioic acid	1.78	1.29E-04	DOWN	1.70	5.19E-04	DOWN	2.10	8.65E-04	UP
Mannitol	1.89	4.46E-07	DOWN	1.96	5.38E-09	DOWN	1.92	5.10E-03	UP
Glucitol	1.89	4.46E-07	DOWN	1.96	5.38E-09	DOWN	1.92	5.10E-03	UP
Galactitol	1.89	4.46E-07	DOWN	1.96	5.38E-09	DOWN	1.92	5.10E-03	UP
Iditol	1.89	4.46E-07	DOWN	1.96	5.38E-09	DOWN	1.92	5.10E-03	UP
Betaine	1.93	2.63E-07	DOWN	1.89	1.59E-06	DOWN	2.46	5.49E-07	DOWN
Gluconic acid	1.93	9.27E-09	DOWN	1.97	5.74E-10	DOWN	2.01	2.42E-03	UP
Glutamine	1.91	4.15E-07	DOWN	1.82	1.69E-05	DOWN	2.21	3.67E-04	UP
Kaempferol	1.78	9.99E-06	DOWN	1.92	1.39E-07	DOWN	1.75	2.12E-02	UP
Monobutyl phthalate	1.97	1.18E-04	DOWN	1.95	2.10E-04	DOWN	2.53	1.30E-04	DOWN
Maltobionate	1.96	5.81E-10	DOWN	1.17	3.46E-02	UP	1.89	4.42E-03	UP
Methyl 3-O-methylgallate	1.95	4.15E-08	DOWN	1.95	2.17E-09	DOWN	1.61	2.95E-02	UP
Glutarylcarnitine	1.86	1.15E-06	DOWN	1.93	1.22E-04	UP	2.17	5.07E-04	UP
N-Benzyloxycarbonylglycine	1.37	9.83E-03	DOWN	1.95	3.77E-08	DOWN	1.94	4.42E-03	DOWN
Purine	1.87	2.98E-06	DOWN	1.88	1.12E-06	DOWN	2.09	1.06E-03	UP
Octadecanedioic acid	1.89	4.02E-06	DOWN	1.79	7.93E-05	DOWN	1.55	3.63E-02	DOWN
4-Vinylphenol	1.91	3.76E-07	DOWN	1.78	2.68E-05	DOWN	1.96	3.09E-03	UP
Ferulate	1.94	2.43E-08	DOWN	1.83	1.66E-05	DOWN	2.29	1.06E-04	DOWN
13,14-Dihydro-15-ketotetranorprostaglandin F1.beta.	1.96	1.60E-06	DOWN	1.94	1.59E-07	DOWN	1.87	5.97E-03	UP
2-oxindole-3-acetate	1.57	1.38E-03	DOWN	1.92	1.08E-05	DOWN	2.53	2.24E-05	UP
Pimelic acid	1.90	9.14E-05	DOWN	1.96	8.31E-06	DOWN	2.24	2.37E-04	DOWN
5-Hydroxyindole-3-acetic acid	1.57	1.38E-03	DOWN	1.83	3.54E-05	DOWN	1.76	1.49E-02	DOWN
3,7-Dimethyluric acid	1.93	9.27E-09	DOWN	1.80	5.46E-05	DOWN	1.60	3.19E-02	DOWN
3-Methyladipic acid	1.90	9.14E-05	DOWN	1.83	3.54E-05	DOWN	1.76	1.49E-02	DOWN
NAE(18:1)	1.92	5.60E-08	DOWN	1.97	5.74E-10	DOWN	2.01	2.42E-03	UP
1,3-Dimethyluric acid	1.93	9.27E-09	DOWN	1.80	5.46E-05	DOWN	1.60	3.19E-02	DOWN
Sulfaquinoxaline (sodium salt)	1.82	6.03E-06	DOWN	1.96	7.35E-09	DOWN	2.24	1.82E-03	UP
(12Z)-9,10,11-Trihydroxyoctadec-12-enoic acid	1.91	1.76E-06	DOWN	1.97	5.74E-10	DOWN	2.01	2.42E-03	UP
Nandrolone	1.80	2.11E-03	DOWN	1.91	3.30E-07	DOWN	1.87	9.67E-03	UP

Quinolin-7-ol	1.92	1.02E-07	DOWN	1.87	5.41E-06	DOWN	2.24	1.75E-04	DOWN
9,12-Octadecadiynoic acid	1.78	6.68E-05	DOWN	1.57	4.71E-03	DOWN	2.01	1.27E-02	DOWN
2-(Hydroxymethyl)-6-methylpyridin-3-ol	1.57	8.32E-04	DOWN	1.52	2.67E-03	DOWN	2.28	4.24E-04	DOWN
Diosmetin	1.80	1.27E-05	DOWN	1.45	6.12E-03	DOWN	2.48	1.34E-07	DOWN
Quinoline-2,6-diol	1.84	2.02E-05	DOWN	1.86	9.60E-06	DOWN	2.16	5.22E-04	DOWN
3,5-Dihydroxy-4-methylbenzoic acid	1.92	2.42E-07	DOWN	1.90	5.30E-07	DOWN	2.00	3.72E-03	UP
PA(14:0/0:0)	1.62	1.22E-03	DOWN	1.88	1.82E-04	DOWN	2.32	9.69E-04	DOWN
5,7,2'-Trihydroxyflavone	1.73	1.55E-04	DOWN	1.98	4.60E-07	UP	1.51	3.95E-02	DOWN
6-(4-Methyl-1-piperazinyl)-N-(5-methyl-1H-pyrazol-3-yl)-2-[(1E)-2-phenylethenyl]-4-pyrimidinamine	1.14	4.41E-02	DOWN	1.96	7.18E-06	DOWN	1.78	9.93E-03	UP
Arg-Pro-Pro	1.94	7.98E-06	DOWN	1.41	1.70E-02	DOWN	2.09	1.27E-03	DOWN
Emodin	1.79	2.93E-05	DOWN	1.90	8.88E-05	DOWN	1.64	2.35E-02	UP
2,2'-Dihydroxy-4,4'-dimethoxybenzophenone	1.91	7.06E-07	DOWN	1.46	1.49E-02	DOWN	1.60	3.01E-02	DOWN
4,5-Dimethoxy-2,3-dihydro-1H-isoindole-1,3-dione	1.18	3.95E-02	DOWN	1.86	1.67E-05	UP	1.58	3.51E-02	UP
Succinic semialdehyde	1.80	6.72E-05	DOWN	1.95	1.40E-05	DOWN	1.64	2.29E-02	DOWN
N-(4-Methyl-2-pyrimidinyl)-1,3-benzoxazol-2-amine	1.63	4.67E-04	DOWN	1.90	3.59E-06	DOWN	1.73	1.56E-02	UP
17-phenyl-trinor-PGE2	1.83	1.51E-05	DOWN	1.76	5.99E-05	DOWN	2.41	6.75E-06	DOWN
3-[3-(2-Piperidinoethoxy)phenyl]-5-(1H-1,2,4-triazol-5-yl)-1H-indazole	1.70	4.09E-04	DOWN	1.63	9.00E-04	DOWN	2.46	1.25E-06	UP
9Z,11E,13E-Octadecatrienoic acid	1.70	6.30E-04	DOWN	1.98	4.86E-07	UP	1.51	4.98E-02	DOWN
[2,2-bis(2-methylpropoxy)ethyl]benzene	1.74	1.14E-04	DOWN	1.61	9.60E-04	DOWN	1.85	7.77E-03	UP
4-Hexylresorcinol	1.82	1.56E-05	DOWN	1.75	4.83E-05	DOWN	1.47	4.98E-02	UP
12,13-DiHOME	1.39	2.47E-03	DOWN	1.56	1.38E-03	DOWN	2.45	1.06E-06	DOWN
4-Methyl-3-(trifluoromethyl)-1H-pyrazol-5-amine	1.91	5.63E-07	DOWN	1.44	6.09E-03	DOWN	2.39	1.36E-05	DOWN
LPI(18:1)	1.55	2.31E-03	DOWN	1.80	4.43E-05	DOWN	2.08	1.14E-02	UP
13,14-Dihydro-15-keto-PGE2	1.82	2.94E-05	DOWN	1.79	4.57E-05	DOWN	2.17	5.63E-04	UP
12,13-Dihydroxy-9Z-octadecenoic acid	1.83	2.46E-03	DOWN	1.66	4.49E-04	DOWN	2.49	9.96E-08	DOWN
N-Acetyl-D-galactosaminitol	1.83	2.43E-07	DOWN	1.74	3.59E-05	DOWN	2.32	2.62E-05	UP

.beta.-Peltatin	1.76	1.11E-05	DOWN	1.94	2.91E-07	DOWN	2.03	1.64E-03	UP
5-(4-Aminophenyl)-4-phenyl-2,4-dihydro-3H-1,2,4-triazole-3-thione	1.29	2.30E-02	DOWN	1.92	9.87E-08	DOWN	1.81	1.17E-02	UP
9(10)-Epoxy-12Z-octadecenoic acid	1.57	3.43E-03	DOWN	1.52	2.75E-03	DOWN	2.46	4.52E-05	DOWN
Sinapic_acid	1.86	9.00E-06	DOWN	1.84	2.13E-03	DOWN	2.26	4.86E-03	DOWN
Methyl 9H-purine-6-carboxylate	1.94	2.64E-08	DOWN	1.89	2.77E-07	DOWN	2.22	2.30E-04	UP
2-(Hydroxymethyl)-5-methyl-4H,7H-[1,2,4]triazolo[1,5-a]pyrimidin-7-one	1.93	2.91E-07	DOWN	1.79	1.47E-05	DOWN	2.37	1.32E-05	DOWN
2,6-Di-tert-butyl-4-ethylphenol	1.29	2.11E-02	DOWN	1.49	4.33E-03	DOWN	1.72	1.74E-02	DOWN
3-Coumaric acid	1.92	3.42E-07	DOWN	1.79	1.42E-04	DOWN	2.17	6.67E-04	UP
DG(18:4(6Z,9Z,12Z,15Z)/18:1(11Z)/0:0)	1.76	9.66E-05	DOWN	1.92	5.10E-07	DOWN	2.49	9.44E-05	UP
9-Oxo-11-(3-pentyl-2-oxiranyl)-10E-undecenoic acid	1.88	5.80E-06	DOWN	1.96	6.86E-09	DOWN	1.89	5.36E-03	UP
16-Hydroxy-10-oxohexadecanoic_acid	1.77	6.37E-05	DOWN	1.91	6.82E-07	DOWN	1.91	5.11E-03	UP
9S,12S,13S-trihydroxy-10E-octadecenoic_acid	1.91	1.14E-06	DOWN	1.73	1.36E-04	DOWN	2.31	1.11E-03	UP
4-Fluoro-.alpha.-pyrrolidinobutiophenone	1.59	1.72E-03	DOWN	1.95	2.68E-05	DOWN	1.99	2.26E-03	UP
2-Naphthalenethiol	1.94	4.03E-09	DOWN	1.86	1.58E-05	DOWN	2.37	1.04E-05	DOWN
2-Carboxybenzeneboronic acid	1.96	6.29E-10	DOWN	1.93	1.37E-04	DOWN	2.08	2.25E-03	DOWN
2-Deoxyribose 5-phosphate	1.55	3.45E-10	DOWN	1.81	4.69E-05	DOWN	2.26	1.11E-04	UP
9-Oxo-9H-fluorene-2-carboxylic acid	1.57	2.21E-03	DOWN	1.89	3.21E-06	DOWN	1.87	1.60E-02	DOWN
8R-HpODE	1.57	1.80E-03	DOWN	1.78	9.32E-05	UP	1.64	2.52E-02	DOWN
3-[(4-Fluoroanilino)carbonyl]-1,2,2-trimethylcyclopentanecarboxylic acid	1.89	1.44E-06	DOWN	1.78	1.05E-04	DOWN	1.79	1.17E-02	DOWN
5-[5-(Acetyloxymethyl)-1,2,4a-trimethyl-7-oxo-3,4,8,8a-tetrahydro-2H-naphthalen-1-yl]-3-methylpentanoic acid	1.73	2.53E-04	DOWN	1.95	1.89E-09	DOWN	1.88	6.31E-03	UP

3-Hydroxysebacic_acid	1.54	3.85E-03	DOWN	1.96	4.29E-09	DOWN	2.45	1.23E-06	DOWN
(S)-3,5-Dihydroxyphenylglycine	1.75	1.26E-04	DOWN	1.63	2.61E-07	DOWN	1.51	3.77E-02	UP
3-(Difluoromethoxy)-4-methoxybenzoic acid	1.95	1.65E-09	DOWN	1.53	3.73E-03	DOWN	1.54	4.14E-02	DOWN
N-Oleoylglycine	1.47	1.03E-03	DOWN	1.96	7.44E-06	UP	1.60	4.34E-02	UP
14-(hydroxymethyl)-5,9-dimethyltetracyclo[11.2.1.0,.0,]hexadecane-5-carbaldehyde	1.94	4.45E-08	DOWN	1.59	1.73E-03	DOWN	2.20	3.65E-03	DOWN
3-(Trifluoromethyl)cinnamic acid	1.92	5.32E-08	DOWN	1.83	1.58E-06	DOWN	2.05	1.61E-03	UP
Catechin 7-apioside	1.92	2.72E-07	DOWN	1.61	1.31E-03	DOWN	2.47	5.93E-07	DOWN
4,8_dimethylnonanoyl_carnitine	1.59	6.22E-03	DOWN	1.16	4.25E-02	DOWN	1.58	3.24E-02	DOWN
1,1,1-Trifluoro-4-(4-hydroxyphenyl)butan-2-one	1.85	5.88E-07	DOWN	1.65	6.40E-04	DOWN	1.76	1.30E-02	DOWN
Vulgarole	1.36	2.97E-02	DOWN	1.97	3.80E-10	DOWN	1.63	2.55E-02	UP
(2-{[2-[hexadec-9-enoyloxy]-3-(tetradecanoyloxy)propyl phosphonato]oxy}ethyl)trimethylazanium	1.55	9.61E-04	DOWN	1.88	4.16E-04	DOWN	1.74	2.74E-03	DOWN
9S,11R-Dihydroxy-15-oxo-13E-prostenoic acid	1.37	1.60E-02	DOWN	1.83	9.79E-06	DOWN	2.41	1.83E-06	DOWN
2,3,4',5-Tetrahydroxystilbene 2-glucoside	1.81	7.63E-05	DOWN	1.92	8.08E-08	DOWN	1.66	2.75E-02	UP
Albocycline	1.35	1.35E-02	DOWN	1.80	5.12E-05	UP	1.93	3.56E-03	DOWN
Poliothyrsoside	1.59	6.33E-04	DOWN	1.93	3.09E-07	DOWN	2.35	3.18E-05	DOWN
1,2-Dimyristoyl-sn-glycero-3-phosphate	1.58	2.11E-03	DOWN	1.28	2.04E-02	DOWN	2.28	1.15E-04	DOWN
2,3-Dinorthromboxane B1	1.95	6.67E-06	DOWN	1.88	8.49E-08	DOWN	2.17	8.32E-04	UP
Hexamethylquercetagetin	1.86	6.18E-06	DOWN	1.60	1.19E-03	DOWN	2.13	8.86E-04	DOWN
1-Nitro-4-(1,1,2,2-tetrafluoroethoxy)benzene	1.69	4.40E-04	DOWN	1.46	3.65E-03	DOWN	2.46	7.47E-07	DOWN
Methyl 1-hydroxy-2-naphthoate	1.73	3.20E-04	DOWN	1.81	8.78E-04	DOWN	2.24	1.70E-04	UP
Antibiotic FR 901512	1.27	3.25E-02	DOWN	1.83	5.30E-04	DOWN	2.26	2.38E-04	DOWN
(5E)-2-Heptyl-3,4,7-trihydroxy-2,3,4,7,8,9-hexahydrooxecin-10-one	1.85	9.99E-06	DOWN	1.50	6.12E-03	DOWN	1.60	3.13E-02	DOWN
Acetylsyringic acid	1.51	3.09E-03	DOWN	1.60	1.46E-03	DOWN	2.03	7.77E-04	DOWN
2-Methoxy-N-(1H-tetraazol-5-yl)benzamide	1.17	4.23E-02	DOWN	1.92	2.73E-05	UP	1.97	2.03E-03	DOWN

Trospium	1.81	4.05E-03	DOWN	1.35	1.72E-02	UP	2.19	4.28E-04	UP
7-((6-Deoxy-.alpha.-L-mannopyranosyl)oxy)-5-hydroxy-2-(4-hydroxyphenyl)-4-oxo-4H-chromen-3-yl 3-O-acetyl-6-deoxy-.alpha.-L-mannopyranoside	1.85	4.32E-06	DOWN	1.76	1.08E-04	DOWN	1.80	1.60E-02	DOWN
5,7-Dihydroxy-1-((2-O-((2E)-3-(4-hydroxyphenyl)prop-2-enoyl)hexopyranosyl)oxy)-7-methyl-1,4a,5,6,7,7a-hexahydrocyclopenta[c]pyran-4-carboxylic acid	1.90	2.56E-07	DOWN	1.94	1.90E-05	DOWN	2.38	2.87E-05	DOWN
1-(2,4-Dinitroanilino)-1H-pyrrole-2,5-dione	1.91	3.11E-07	DOWN	1.92	3.49E-07	DOWN	1.63	2.50E-02	UP
Specioside	1.81	3.01E-05	DOWN	1.75	2.13E-03	DOWN	2.16	9.33E-04	DOWN
3-[(4-Phenyl-1-phthalazinyl)amino]-1-propanol	1.49	1.03E-02	DOWN	1.81	2.02E-03	DOWN	2.14	1.41E-03	DOWN
Harman	1.90	1.17E-07	DOWN	1.95	1.62E-05	UP	1.89	6.54E-03	UP
Fenpyroximate (Z,E)	1.82	1.99E-06	DOWN	1.56	1.56E-03	DOWN	2.17	2.90E-04	DOWN
3-(3,5-Difluorophenyl)-L-alanine	1.71	5.89E-04	DOWN	1.83	3.32E-05	DOWN	1.70	1.70E-02	DOWN
1-(Chloromethyl)-2,8,9-trioxa-5-aza-1-silabicyclo[3.3.3]undecane	1.91	4.25E-07	DOWN	1.28	2.63E-02	UP	2.01	2.71E-03	UP
9,10-Dihydroxystearate	1.97	1.44E-10	DOWN	1.76	2.19E-04	UP	1.77	1.47E-02	UP
M351T260	1.85	2.05E-05	DOWN	1.24	3.02E-02	DOWN	1.81	9.71E-03	DOWN
M367T229	1.77	1.14E-04	DOWN	1.91	1.03E-04	UP	1.86	7.00E-03	UP
M459T300	1.49	8.06E-04	DOWN	1.28	1.73E-02	DOWN	1.79	2.31E-02	DOWN
M361T251	1.81	2.88E-06	DOWN	1.11	2.25E-04	UP	1.06	4.54E-02	UP
Histidine	1.36	5.31E-03	UP	1.58	5.80E-03	DOWN	2.10	9.18E-03	DOWN
Glycerophosphocholine	1.95	5.34E-06	UP	1.71	6.77E-04	UP	1.67	3.17E-02	UP
4-(3,4-Dihydroxyphenyl)-7-methoxy-2-oxo-2H-chromen-5-yl .beta.-D-glucopyranoside	1.96	5.77E-05	UP	1.70	4.02E-05	DOWN	2.10	8.86E-04	DOWN
6,7-Dimethylesculetin	1.70	6.38E-03	UP	1.93	1.18E-08	DOWN	2.11	1.14E-03	UP
Oenin	1.94	8.19E-04	UP	1.95	6.70E-08	DOWN	1.66	2.02E-02	UP
Licochalcone A	1.68	9.11E-04	UP	1.17	4.30E-02	DOWN	2.39	5.95E-06	DOWN
N-Acetylsulfamethoxazole	1.94	3.63E-05	UP	1.84	4.86E-05	UP	1.49	3.75E-02	UP

gamma-Eudesmol_rhamnoside	1.81	7.85E-06	UP	1.92	4.55E-08	DOWN	2.16	1.06E-03	UP
Epigallocatechin	1.54	7.41E-03	UP	1.89	4.80E-08	DOWN	2.04	1.96E-03	UP
N-Palmitoylglycine	1.75	7.20E-05	UP	1.93	3.79E-08	DOWN	2.26	5.23E-05	DOWN
Echinulin	1.90	1.18E-06	UP	1.75	2.56E-03	DOWN	2.18	9.84E-04	DOWN
Flavaspidic acid AB	1.50	3.60E-03	UP	1.92	1.82E-07	DOWN	1.83	7.87E-03	DOWN
Prohexadione	1.70	4.54E-04	UP	1.42	8.09E-03	UP	1.57	2.81E-02	DOWN
4-Acetyl-2-methoxyphenyl 6-O-(6-deoxy-.alpha.-L-mannopyranosyl)-.beta.-D-glucopyranoside	1.91	1.26E-06	UP	1.97	2.19E-10	DOWN	2.51	3.52E-09	DOWN
delta-Tocotrienol	1.21	5.47E-05	UP	1.51	1.74E-02	UP	1.52	3.72E-02	UP
Angoroside A	1.47	4.25E-02	UP	1.86	1.08E-05	DOWN	2.23	2.24E-04	DOWN
6-O-Methylarmillaridin	1.89	2.00E-06	UP	1.41	6.34E-03	DOWN	2.35	1.63E-05	DOWN
Cyclo(prolyltyrosyl)	1.74	3.67E-04	UP	1.70	1.58E-04	DOWN	1.87	5.44E-03	UP
(24R,24(1)R)-Fucosterol epoxide	1.68	9.38E-03	UP	1.81	7.28E-07	DOWN	2.16	1.00E-04	DOWN
M353T152	1.93	1.42E-05	UP	1.95	3.16E-08	UP	1.65	2.59E-02	UP

Table S1. Differential metabolites in rice grains under four storage conditions. VIP denoted the degree to which the metabolite contributes to the sample's classification. P denoted the *p*-value from the Student's t-test.
